# Mild Oxidative Stress Reduces NRF2 SUMOylation to Promote *Kras*/*Lkb1*/*Keap1* Mutant Lung Adenocarcinoma Cell Migration and Invasion

**DOI:** 10.1155/2020/6240125

**Published:** 2020-11-24

**Authors:** Jiaqian Xu, Haoyan Guo, Zhengcao Xing, Wenlong Zhang, Jianli He, Jinke Cheng, Rong Cai

**Affiliations:** ^1^Department of Biochemistry & Molecular Cell Biology, Shanghai Jiao Tong University School of Medicine, Shanghai 200025, China; ^2^Department of Pathology, Beijing Obstetrics and Gynecology Hospital, Capital Medical University, Beijing 100026, China; ^3^Department of Cell Biology, Shaanxi Normal University, Xi'an 710062, China

## Abstract

Nuclear factor erythroid 2-related factor 2 (NRF2) is a crucial transcription factor for cell adaptation and defense against oxidative stress. NRF2 activation confers *Kras*/*Lkb1*/*Keap1* (KLK) mutant tumor cells with greater resistance to oxidative insults. We previously reported that SUMOylation at lysine residue 110 is important for the ability of NRF2 to promote reactive oxygen species (ROS) clearance in hepatocellular carcinoma. In this study, we investigated whether SUMOylation is necessary for the ability of NRF2 to inhibit KLK lung adenocarcinoma (LUAD) cell migration and invasion. Our experiments showed that mild oxidative stress reduced NRF2 SUMOylation, which promoted KLK LUAD cell migration and invasion. Mechanistically, NRF2 SUMOylation increased the antioxidant ability of NRF2 and reduced cellular ROS levels, mainly by transcriptionally activating *Cat* in KLK LUAD cells. With reduced NRF2 SUMOylation, increased ROS acted as signaling molecules to activate the JNK/c-Jun axis, which enhanced cell mobility and cell adhesion, to promote LUAD cell migration and invasion. Taken together, the results of this study reveal a novel signaling process in which reduced NRF2 SUMOylation permits increased KLK LUAD cell migration and invasion under mild oxidative stress.

## 1. Introduction

The role of reactive oxygen species (ROS) in cancer has remained controversial for decades, in part, because different levels of ROS confer different outcomes in cancer cells. High ROS levels are harmful to cell, but mild oxidative stress at sublethal levels activates signaling pathways to promote tumor growth and progression [1, 2]. Cancer cell migration and invasion are the initial steps of tumor metastasis. During cell migration and invasion, members of the mitogen-activated protein kinase (MAPK) family of proteins are activated by ROS [3–5]. In lung adenocarcinoma cells (LUAD), H_2_O_2_ activates epidermal growth factor (EGF) receptors [6]; hence, oxidization of receptor tyrosine kinases (RTKs) facilitates MAPK signaling activation and promotes migration and invasion [7].

In *Kras*-mutant LUAD, *Lkb1* is frequently inactivated [8–11]. LKB1 loss leads to increased oxidative stress in tumors [12, 13], which is tolerated at least partially through concurrent mutation of KEAP1 [11, 14]. KEAP1 mutation stabilizes nuclear factor erythroid 2-related factor 2 (NRF2) and increases its activity in LUAD [15, 16]. NRF2 is an important transcription factor in the defense of cancer cells against oxidative insults, through upregulation of antioxidant enzymes and detoxification proteins [17]. Thus, NRF2 activity is critical for reducing cellular ROS levels and maintaining redox homeostasis.

Previous research showed that drugs used in type 2 diabetes mellitus activate nuclear factor erythroid 2-related factor 2 (NRF2) and accelerate metastasis [18]. Recently, concurrent studies by two research groups demonstrated that activation of NRF2 caused by KEAP1 inactivation promotes LUAD cell migration and metastasis by stabilizing the transcription factor BACH1, in *Kras*^LSL/+^; *Trp53*^*flox*/flox^ (KP) mice [19, 20]. However, the role of NRF2 activation in *Kras*/*Lkb1*/*Keap1* (KLK) mutant LUAD cell migration and metastasis remains unknown.

Multiple studies have reported that NRF2 is a SUMOylated protein [21–23]. Our previous research revealed that SUMOylation of lysine residue 110 (K110) of NRF2 reduces ROS levels, promotes *de novo* serine synthesis, and maintains hepatocellular carcinoma tumorigenesis [23]. In the present study, we investigated the effects of NRF2 on KLK LUAD cell migration and invasion, and whether SUMOylation is critical for these effects. We studied the effect of mild oxidative stress on NRF2 SUMOylation and then investigated the underlying mechanism by which NFR2 influences KLK LUAD cell migration and invasion.

## 2. Materials and Methods

### 2.1. Antibodies, Plasmids, and Reagents

The sources for antibodies were as follows: NRF2 (Abcam; ab62352), BACH1 (R&D Systems; AF5776-SP), Catalase (Abcam; ab76024), GPX2 (GeneTex; GTX100292), SAPK/JNK (Cell Signaling Technology; 9252), phospho-SAPK/JNK (Thr183/Tyr185) (Cell Signaling Technology; 4668), ERK1+ERK2 (Abcam; ab36991), ERK1 (pT202/pY204)+ERK2 (pT185/pY187) (Abcam; ab50011), c-Jun (Cell Signaling Technology; 9165), Phospho-c-Jun (Ser73) (Cell Signaling Technology; 3270), p38 MAPK (Cell Signaling Technology; 9212), phospho-p38 MAPK (Thr180/Tyr182) (Cell Signaling Technology; 4511), His (Qiagen; 1007598), and *β*-actin (Abcam; ab8226). Plasmids PCDH-Vector, PCDH-NRF2, and PCDH-NRF2 K110R were constructed as previously reported [23]; PCDH-His-SUMO1 was cloned into PCDH-vector using standard PCR-based cloning strategies and the primers listed in Table S1. The shNRF2 lentivirus was designed and packaged by Genomeditech as previously reported [23]. The SimpleChIP® Plus Sonication Chromatin IP Kit was purchased from Cell Signaling Technology; the Amplite™ Colorimetric Hydrogen Peroxide Assay Kit was purchased from AAT Bioquest; the eBioscience™ Annexin V Apoptosis Detection Kit was purchased from Invitrogen; the GSH and GSSG Assay Kit was purchased from Beyotime Biotechnology; and the CellROX® Deep Red Flow Cytometry Assay Kit was purchased from Invitrogen.

### 2.2. Cell Culture and Construction of Stable Cell Lines

The human LUAD cell line A549 was cultured in F12K medium (Gibco), while H2122 and H23 cells were cultured in RPMI medium (HyClone). Each medium contained 10% fetal bovine serum (Gibco) and 1% penicillin/streptomycin. These cells were cultured in a humidified 37°C incubator with 5% CO_2_. Cells were infected by shNRF2 and/or PCDH-NRF2/NRF2 K110R lentivirus and then selected by Blasticidin S or Puromycin for 1 week.

### 2.3. Western Blotting

Cells were washed with phosphate-buffered saline (PBS), lysed in radio immunoprecipitation assay (RIPA) buffer (150 mM NaCI, 50 mM Tris base, 0.1% SDS, 1% Triton-X-100, pH 7.4) on ice for 20 min, and then ultrasonicated until the solution became clear. Protease and phosphatase inhibitors were added to the RIPA buffer in advance. The cell lysates were centrifuged at 12,000 rpm for 15 min at 4°C, and the supernatant was collected for Western blotting. Total protein was resolved in 10% SDS/PAGE gels, followed by electrophoretic transfer to PVDF membranes in a Tris-glycine buffer. The membranes were blocked at room temperature for 1 h in 5% nonfat milk with TBS-Tween (TBS-T) on a shaker and then incubated with the primary antibodies overnight at 4°C. The membranes were washed in TBST at least 5 times (5 min each) and then incubated with HRP conjugated anti-rabbit or anti-mouse IgG at room temperature for 1 h with gentle shaking. The ECL substrate was added, and the results were visualized using ImageQuant LAS 4000 (GE). *β*-Actin was used as loading control.

### 2.4. Quantitative Real-Time PCR

Total RNA was isolated using TRIzol universal reagent (TianGen). Then, 1 *μ*g RNA was reverse transcribed into complementary DNA (cDNA) using the Fast King gDNA Dispelling RT SuperMix kit (TianGen). Quantitative real-time PCR was performed on LightCycler 480 (Roche) using TB Green Premix (Takara). 18S rRNA was used as a control for normalization. The primers used in this study are listed in Supplementary Table S1.

### 2.5. Migration and Invasion Assay

For the migration assay, cells were resuspended in serum-free medium and plated in the upper chamber of Transwells (Corning) in a 24-well plate. The cell numbers were 1 × 10^4^ to 1 × 10^5^ per well. For the invasion assay, Transwells were coated with Matrigel matrix (Corning). Medium with 10% fetal bovine serum (FBS) was added in the bottom chamber of the Transwells. The cells on the upper surface of the chamber were removed using a cotton swab, and the cells on the bottom of the chamber were fixed with 4% paraformaldehyde and stained with crystal violet 12-16 h later. For H_2_O_2_ treatment, 0.5, 50, or 500 *μ*M H_2_O_2_ was added into the upper chamber. The migrating and invading cells were counted in micrographs taken of 5 random fields using Photoshop, and the data were analyzed using Graphpad Prism.

### 2.6. NRF2 SUMOylation by Ni^2+^-NTA Pull-down Assay

Cells were infected with PCDH-His-SUMO1 lentivirus and selected by puromycin for 1 week. Once the cells reached 70% confluency, they were treated with H_2_O_2_ (0.5, 50, or 500 *μ*M) for 12 h. For NAC (N-acety-L-cysteine) treatment, cells were pretreated with 1 mM NAC for 1 h, and then, H_2_O_2_ was added for further incubation for 12 h. Ni^2+^-NTA pull-down assay was performed as previously reported [23].

### 2.7. Measurement of Intracellular ROS

Cells were digested by trypsin and incubated with CellROX (final concentration of 100 nM; Invitrogen) in complete medium for 20 min at 37°C. Cells were then washed and resuspended in fluorescence-activated cell sorting (FACS) buffer. Intracellular ROS levels were measured by flow cytometry (Becton Dickinson).

### 2.8. Measurement of H_2_O_2_

Cells were resuspended in a 96-well plate, and the H_2_O_2_ concentration in each well was measured using the Amplite Colorimetric Hydrogen Peroxide Assay Kit (AAT Bioquest) the next day. Briefly, 50 *μ*l of H_2_O_2_ cell working solution was added to each well of cells and H_2_O_2_ standards to make the total H_2_O_2_ assay volume of 100 *μ*l/well. The reaction was incubated at room temperature for 10-60 min and protected from light. Then, the absorbance at 650 nm was measured by an absorbance plate reader.

### 2.9. RNA-Seq

RNA-Seq was performed by KangChen Bio-tech, Shanghai, China. Briefly, the RNA-seq library was prepared using Illumina kits. The sequencing was performed using Illumina Hiseq 4000. Sequencing was carried out by running 150 cycles. Principal component analysis (PCA), hierarchical clustering, correlation analysis, pathway analysis, and gene ontology (GO) were performed, and volcano plots and scatter plots were generated to identify the differentially expressed genes using R or Python environment for statistical computing and graphics.

### 2.10. Measurement of GSH and GSSG

Total glutathione (GSH+GSSG) and oxidized glutathione disulfide (GSSG) were measured using a GSH and GSSG Assay Kit (Beyotime) according to the manufacturer's instructions. The GSH/GSSG ratio was then calculated.

### 2.11. Chromatin Immunoprecipitation Assay

The chromatin immunoprecipitation (ChIP) assay was performed using the Simple ChIP Plus Sonication Chromatin IP Kit (Cell Signaling Technology) according to the manufacturer's protocol. Briefly, cells were fixed with formaldehyde, and the chromatin was sheared with sonication into 200–1000 bp DNA-protein fragments. Then, the NRF2 antibody (or IgG antibody as a control) was added. The complex was coprecipitated and captured by Protein G beads. The protein-DNA cross-links were reversed, and the DNA was purified. Then, the enrichment of the *Cat* promoter was detected by RT-PCR. The primers used in this assay are listed in Supplementary Table S2.

### 2.12. Statistical Analysis

All results were obtained from at least three independent experimental replicates and are presented as mean ± standard error of the mean (S.E.M.). Significance was determined by Student's *t* test. *P* < 0.05 was considered significant.

## 3. Results

### 3.1. SUMOylation of NRF2 Is Critical for Its Inhibition of KLK LUAD Cell Migration and Invasion

As expected, NRF2 expression was significantly lower in *Kras*/*Lkb1* (KL) mutant LUAD H23 cells than in KLK LUAD A549 and H2122 cells (Figure S1A). Correspondingly, H23 cells showed a higher intracellular ROS level than A549 and H2122 cells did (Figure S1B). Upon knockdown of NRF2, the ROS levels in A549 and H2122 cells were elevated, whereas the ROS level in H23 cells was not altered (Figure S1C and S1D). Additionally, KLK LUAD cell migration and invasion were increased with NRF2 knockdown (Figure S1E), revealing that NRF2 inhibits KLK LUAD cell migration and invasion. In contrast, NRF2 knockdown in H23 cells delayed cell migration and invasion (Figure S1E), which is consistent with previous studies showing that downregulation of BACH1 inhibits lung cancer metastasis [19, 20]. However, in the KLK LUAD cell lines, BACH1 expression was not affected (Figure S1F), indicating that NRF2 regulates KLK LUAD migration and invasion independent of BACH1.

**Figure 1:**
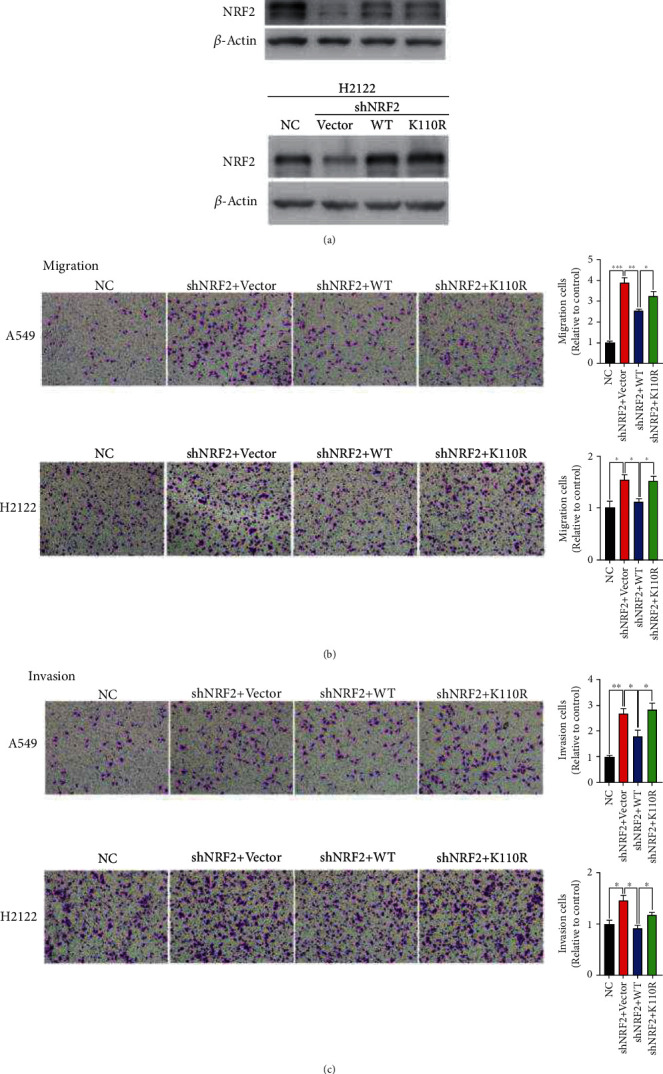
SUMOylation of NRF2 is critical for its inhibition of KLK LUAD cell migration and invasion. (a) Establishment of KLK LUAD A549 and H2122 cells, which have endogenous NRF2 knockdown, stably expressing NRF2 wild-type, and NRF2 K110R. (b) The migration of four stable cell lines derived from A549 and H2122 cells, as detected by Transwell assays (3 replicates per group). (c) The invasion of four stable cell lines derived from A549 and H2122 cell as detected by Transwell assay (3 replicates per group). KLK: *Kras*-activated mutation, *Lkb1*-inactivated mutation, and *Keap1*-inactivated mutation; LUAD: lung adenocarcinoma. ∗*P* < 0.05, ∗∗*P* < 0.01, and ∗∗∗*P* < 0.001.

To investigate the role of NRF2 and its SUMOylation in KLK cell migration and invasion, we restored NRF2 wild-type and NRF2 with SUMOylation site mutation (K110R) expression individually in A549 and H2122 cells (Figure 1(a)). Expression of NRF2 wild-type inhibited cell migration and invasion, which had been enhanced by NRF2 knockdown, but NRF2 K110R did not exhibit this inhibitory capability (Figures 1(b) and 1(c)). Although NRF2 SUMOylation promoted KLK LUAD tumorigenesis and did not affect KLK LUAD cell apoptosis (Figure S2), as reported in HCC previously [23], the results in Figure 1 suggest that SUMOylation is critical for the inhibitory effect of NRF2 on KLK cell migration and invasion.

### 3.2. Mild Oxidative Stress Reduces NRF2 SUMOylation to Induce KLK LUAD Cell Migration and Invasion

**Figure 2:**
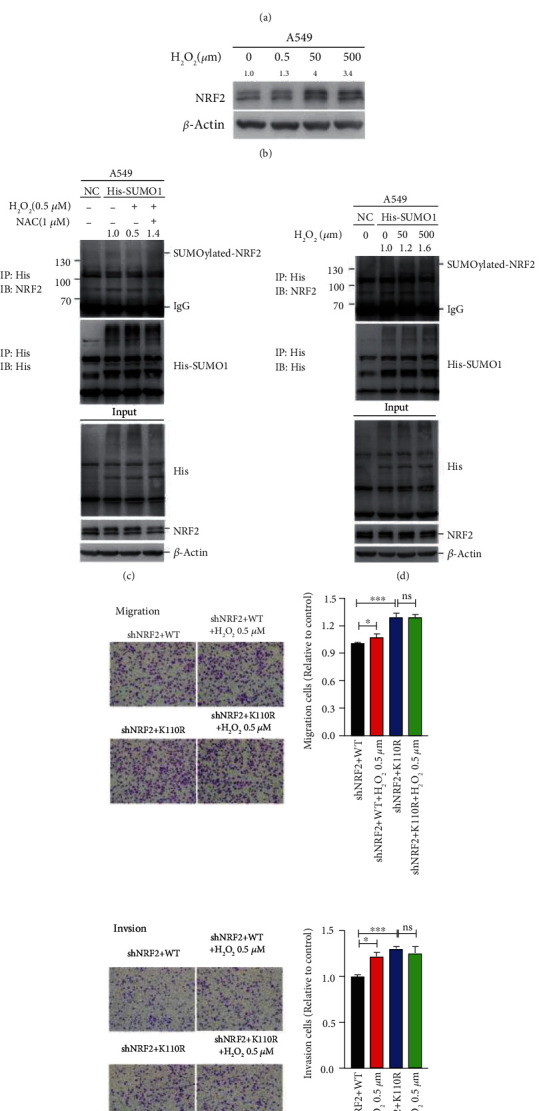
Mild oxidative stress reduces NRF2 SUMOylation to induce KLK LUAD cell migration and invasion. (a) The migration and invasion of A549 cells treated with 0.5, 50, or 500 *μΜ* H_2_O_2_ individually for 12 h (3 replicates per group). (b) NRF2 expression in A549 cells treated with 0.5, 50, or 500 *μΜ* H_2_O_2_ for 12 h. Blots were quantified and normalized to *β*-actin expression. (c) NRF2 SUMOylation in A549-His-SUMO1 cells treated with 0.5 *μΜ* H_2_O_2_ for 12 h. SUMOylated proteins with His tag were purified from cell lysates using Ni^2+^-NTA agarose bead pull-down, and SUMOylated NRF2 was detected by immunoblotting with anti-NRF2 antibody. Blots were quantified and normalized to *β*-actin expression. (d) NRF2 SUMOylation in A549-His-SUMO1 cells treated with 50 or 500 *μΜ* H_2_O_2_ for 12 h. (e) The migration and invasion of A549-shNRF2+WT and A549-shNRF2+K110R cells treated with 0.5 *μΜ* H_2_O_2_ for 12 h (3 replicates per group). H_2_O_2_: hydrogen peroxide. ∗*P* < 0.05, ∗∗*P* < 0.01, and ∗∗∗*P* < 0.001.

Hydrogen peroxide acts as a signaling molecule to induce cancer cell migration and invasion. In A549 cells, we found that mild oxidative stress generated by treatment with 0.5 *μ*M H_2_O_2_ induced cell migration and invasion, whereas moderate/severe oxidative stress (50 *μ*M/500 *μ*M H_2_O_2_) did not induce and even reduced cell migration and invasion (Figure 2(a)). Consistent with previous reports, moderate/severe oxidative stress induced NRF2 expression as a cellular defense response, whereas mild oxidative stress had no impact on NRF2 expression (Figure 2(b)). Because endogenous NRF2 SUMOylation is difficult to detect experimentally, we constructed a His-SUMO1 overexpressing stable cell line named A549-His-SUMO1 and pulled down SUMO1-modified proteins with Ni^2+^-NTA. Surprisingly, we detected that mild oxidative stress (0.5 *μ*M H_2_O_2_) reduced NRF2 SUMOylation in A549-His-SUMO1 cells (Figure 2(c)), whereas SUMOylation of NRF2 was increased after treatment with 50 *μ*M or 500 *μ*M H_2_O_2_ given that NRF2 expression was increased (Figure 2(d)). More importantly, the decrease in NRF2 SUMOylation caused by mild H_2_O_2_ (0.5 *μ*M) was reversed when antioxidant NAC (N-acety-L-cysteine) was added (Figure 2(c)). With a deficiency in NRF2 SUMOylation, as shown in shNRF2+K110R A549 cells, mild oxidative stress no longer induced cell migration and invasion (Figure 2(e)), indicating that mild oxidative stress in KLK LUAD cells induces cell migration and invasion by reducing NRF2 SUMOylation.

### 3.3. SUMOylation Is Critical for the Antioxidant Ability of NRF2 via Transcriptional Activation of Cat in KLK LUAD Cells

**Figure 3:**
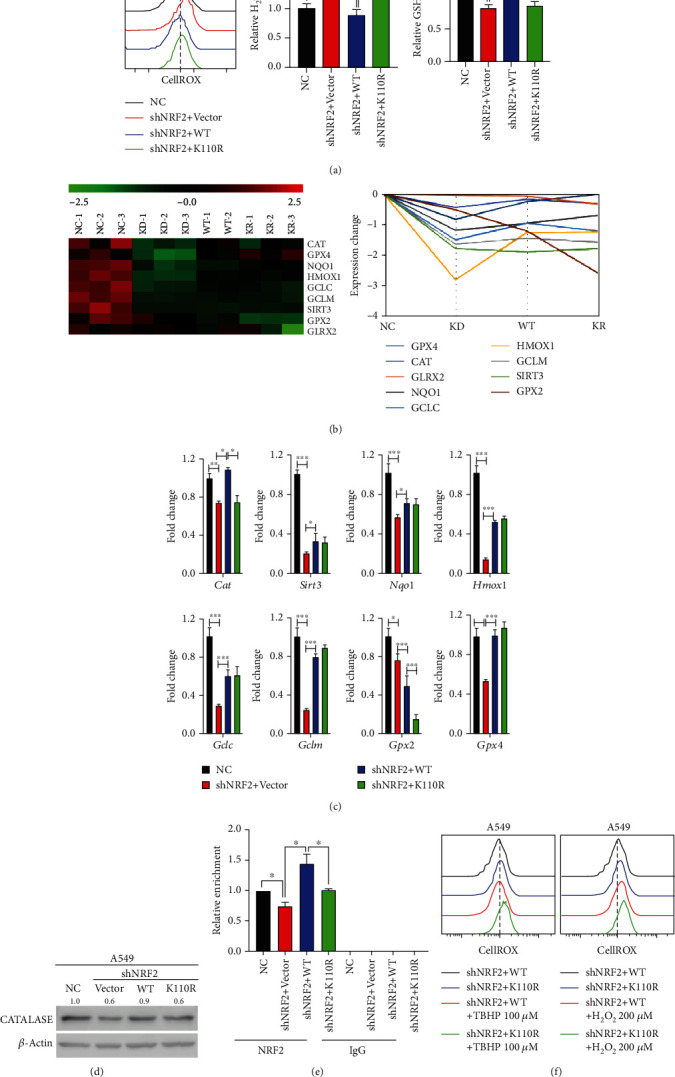
SUMOylation of NRF2 is critical for its antioxidant ability in KLK LUAD *via* transcriptional activation of *Cat*. (a) The intracellular ROS and H_2_O_2_ levels and GSH/GSSG ratio in four stable cell lines constructed from A549 cells (3 replicates per group). (b) Heat map and trend graph comparing the patterns of antioxidant gene expression in four stable cell lines constructed from A549 cells. (c) Validation of gene expression involved in antioxidant pathways by quantitative real-time PCR in four stable cell lines constructed from A549 cells (3 replicates per group). *Cat*: catalase; *Sirt3*: Sirtuin 3; *Nqo1*: NAD(P)H quinone dehydrogenase 1; *Hmox1*: heme oxygenase 1; *Gclc*: glutamate-cysteine ligase catalytic subunit; *Gclm*: glutamate-cysteine ligase modifier subunit; *Gpx2*: glutathione peroxidase 2; *Gpx4*: glutathione peroxidase 4. (d) Catalase protein expression analyzed by Western blotting in four stable cell lines constructed from A549 cells. Blots were quantified and normalized to *β*-actin expression. (e) ChIP assay of NRF2 occupancy in the locus of *Cat* promoter in A549-shNRF2+WT and A549-shNRF2+K110R cells. (f) A549-shNRF2+WT and A549-shNRF2+K110R cells were treated with 100 *μΜ* TBHP or 200 *μΜ* H_2_O_2_ for 12 h. The intracellular ROS level was then measured by flow cytometry. TBHP: tert-butyl hydroperoxide; H_2_O_2_: hydrogen peroxide. ∗*P* < 0.05, ∗∗*P* < 0.01, and ∗∗∗*P* < 0.001.

We previously reported that NRF2 SUMOylation reduces the intracellular ROS level in HCC cells [23]. In A549 cells, NRF2 SUMOylation reduced both the total ROS and H_2_O_2_ levels (Figure 3(a)). In addition, the reduced glutathione (GSH) to oxidized glutathione disulfide (GSSG) (GSH/GSSG) ratio was relatively higher in A549 cells in which NRF2 wild-type expression was rescued (Figure 3(a)). To explore the underlying mechanism, we performed RNA sequencing (RNA-Seq) analysis to compare the gene expression levels in A549 cells before (normal control, NC group) and after NRF2 knockdown (KD group), as well as in A549 cells expressing NRF2 wild-type (WT group) and NRF2 K110R (KR group). Volcano plots showed that 1299 genes were significantly upregulated, and 687 genes were downregulated in the KD group versus NC group (Figure S3A), and 99 genes were significantly upregulated, and 255 genes were downregulated in the KR group versus WT group (Figure S3C). Gene ontology (GO) analyses showed that upon NRF2 knockdown, pathways involved in the response to oxidative stress and the response to hydrogen peroxide were downregulated (Figure S3B). The heat map and trend graph revealed that in A549 cells, NRF2 SUMOylation promotes H_2_O_2_ removal mainly by activating *Cat* transcription, which was validated by our quantitative real-time polymerase chain reaction (PCR) and Western blot results (Figures 3(b)–3(d)). Different from our previous report [23], NRF2 SUMOylation did not alter the protein expression of GPX2 in KLK cells (Figure S3E). ChIP assay showed that NRF2 K110R had a significantly decreased ability to bind with the *Cat* gene promoter (Figure 3(e)). Moreover, upon challenge of A549 cells with oxidants (tert-butyl hydroperoxide [TBHP] and H_2_O_2_), the antioxidant ability of NRF2 was reduced when its SUMOylation site was mutated (Figure 3(f)). These results collectively reveal that SUMOylation is critical for the antioxidant ability of NRF2, mainly *via* the transcriptional activation of *Cat* in KLK LUAD cells.

### 3.4. Increased ROS Level due to NRF2 SUMOylation Deficiency Promotes KLK LUAD Cell Migration and Invasion via JNK/c-Jun Axis

**Figure 4:**
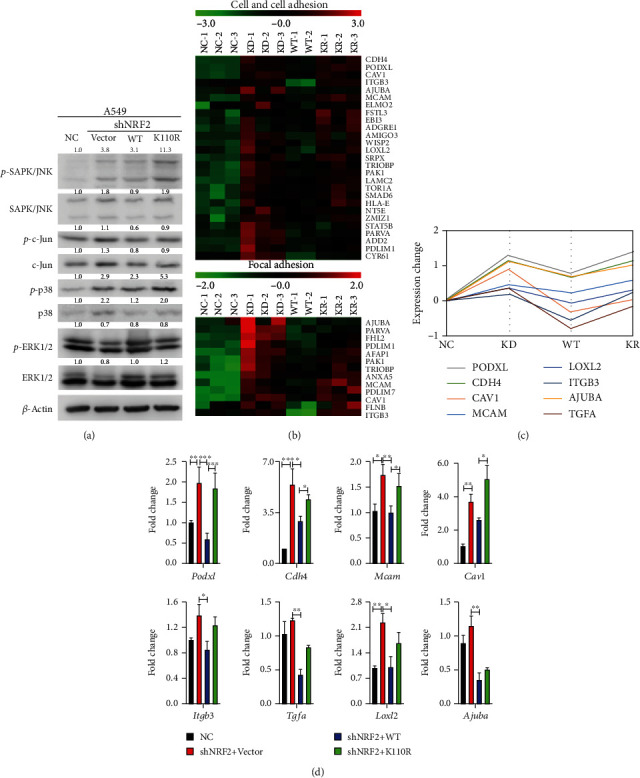
Mild ROS act as signaling molecules to activate the JNK/c-Jun axis and promote KLK LUAD cell migration and invasion. (a) The altered activation of MAPK signaling pathways (ERK, JNK, and p38 signaling pathways) in four stable cell lines constructed from A549 cells. MAPK: mitogen-activated protein kinase; ERK: extracellular signal-regulated kinase; JNK: c-Jun NH-2 terminal kinase. Blots were quantified and normalized to *β*-actin expression. (b) Heat map comparing patterns of gene expression related to cell and cell adhesion as well as focal adhesion in four stable cell lines constructed from A549 cells. (c) Trend graph comparing patterns of gene expression related to cell and cell adhesion as well as focal adhesion in four stable cell lines constructed from A549 cells. (d) Validation of expression of genes involved in cell and cell adhesion as well as focal adhesion by quantitative real-time PCR in four stable cell lines constructed from A549 cells (3 replicates per group). *Podxl*: podocalyxin like; *Cdh4*: cadherin 4; *Mcam*: melanoma cell adhesion molecule; *Cav1*: caveolin 1; *Itgb3*: integrin subunit beta 3; *Tgfa*: transforming growth factor alpha; *Loxl2*: lysyl oxidase like 2; *Ajuba*: Ajuba LIM protein. ∗*P* < 0.05, ∗∗*P* < 0.01, and ∗∗∗*P* < 0.001.

Based on our finding that ROS and H_2_O_2_ levels were increased with deficient NRF2 SUMOylation (Figure 3(a)), we then asked whether increased intracellular ROS act as signaling molecules to activate pathways that promote KLK LUAD cell migration and invasion. As shown in Figure 4(a), when NRF2 expression was inhibited and the intracellular ROS level was increased, the JNK/c-Jun axis was activated in KLK LUAD cells. Expression of NRF2 wild-type inhibited the activation of the JNK/c-Jun axis, whereas expression of NRF2 K110R did not (Figure 4(a)). Furthermore, the results of RNA-Seq and quantitative real-time PCR analyses revealed that through increasing the gene expression related with cell motility and cell adhesion, JNK/c-Jun axis activation promoted the migration and invasion in KLK LUAD cells (Figure S3D and Figures 4(b)–4(d)). In addition, we demonstrated NRF2 SUMOylation inhibited cell migration and invasion by activating *Cat* transcription and inactivating the JNK/c-Jun axis by reducing the cellular ROS level, in KLK LUAD H2122 cells (Figure S4).

## 4. Discussion

In KLK LUAD, KEAP1 mutation leads to NRF2 activation, which influences many of the hallmarks of cancer and confers “NRF2 addition” [24]. High NRF2 activity renders KLK LUAD more resistant to ROS accumulation compared with KL LUAD [14]. In contrast, to promote tumorigenesis, NRF2 SUMOylation inhibits the migration and invasion of KLK LUAD cells (Figure 1), further complicating the anti-versus protumorigenic roles of ROS in cancer cells. The extent of ROS increase often determines the adaptive consequences of the cellular response to the oxidative insult. We revealed in the present study that, in comparison to severe oxidative stress, which inhibited cell migration and invasion, mild oxidative stress promoted the migration and invasion of KLK LUAD cells (Figure 2(a)). SUMOylation of vimentin (VIM), a type III intermediate filament protein involved in cytoskeleton organization and cell motility, favors cell motility and migration [25]. However, whether protein SUMOylation is involved in ROS-triggered cancer cell migration and invasion has remained largely unknown. The results of the present study demonstrate that the SUMOylation of NRF2, an essential antioxidant factor, plays a role in the inhibition of KLK LUAD migration and invasion.

NRF2 is stabilized upon hyperoxidation of cysteines on KEAP1 which disrupts the binding of the two proteins [15, 17]. Here, we revealed that mild oxidative stress reduced NRF2 SUMOylation (Figure 2(c)), rather than increasing NRF2 expression as seen with moderate/severe oxidative stress (Figure 2(b)), suggesting a mechanism by which NRF2 activity is regulated independently of KEAP1. It has been reported that SUMO-specific protease 3 (SENP3) is a redox sensor that is stabilized under mild oxidative stress and consequently de-SUMOyltates p300 and hypoxia inducible factor (HIF)-1*α* [26, 27]. Recently, Zhou et al. reported that NRF2 activity is regulated by SENP3 in laryngeal carcinoma after cisplatin-induced ROS stress [28]. Because SENP1 is also reported de-SUMOylate NRF2 [22], whether mild oxidative stress decreases NRF2 SUMOylation by increasing SENP3 expression or by increasing the expression and/or activities of other SENPs, in KLK LUAD cells, needs to be explored in future experiments.

In the present study, we showed that NRF2 SUMOylation reduced the intracellular ROS level mainly *via* the transcriptional activation of *Cat* to promote H_2_O_2_ removal (Figure 3). We also found that NRF2 SUMOylation reduced the intracellular ROS level by enhancing GPX2 protein expression in KLK LUAD cells (Figure S3C). We found that the increase in the H_2_O_2_ level caused by NRF2 SUMOylation deficiency in A549 cells was in the nanomolar range. Hence, at mildly increased level, H_2_O_2_ acts as a signaling molecule to activate the JNK/c-Jun pathway, which enhances cell-cell adhesion as well as focal adhesion, to promote cell migration and invasion (Figure 4). However, how the level of increased ROS brought about by NRF2 SUMOylation deficiency specifically regulates JNK/c-Jun axis activation in KLK LUAD cells remains to be determined in future research.

A recent report showed that ROS restriction by TIGAR supports premalignant tumor initiation while restricting metastasis in pancreatic ductal adenocarcinoma, indicating that the complexity of ROS regulation underpins full malignant progression [29]. The present study demonstrated that by reducing the ROS level, NRF2 SUMOylation can regulate KLK LUAD progression in a stage-specific manner. Thus, we conclude that NRF2 SUMOylation promotes KLK LUAD tumorigenesis. However, during the initiation stage of tumor metastasis, mildly increased oxidative stress resulting from detachment may reduce NRF2 SUMOylation to induce cell migration and invasion, a potential mechanism that should be investigated *in vivo* in future studies. Our findings herein are helpful in understanding the *Kras*-dependent pathways that promote LUAD progression and provide insights for the identification of potential therapeutic targets and the development of new treatments for KRAS mutant LUAD.

## 5. Conclusions

**Figure 5:**
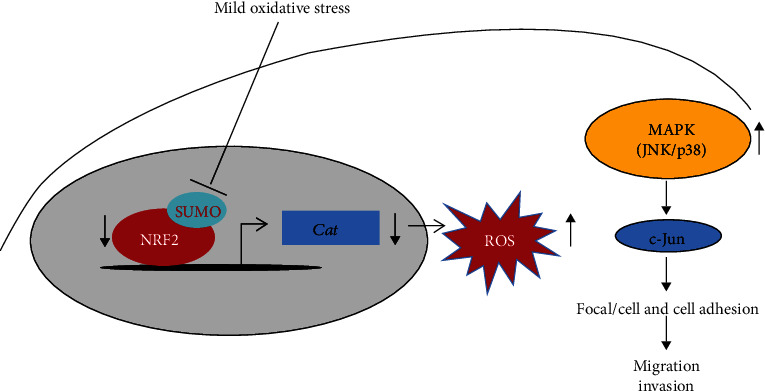
Model depicting how mild oxidative stress reduces NRF2 SUMOylation to promote KLK LUAD migration and invasion.

On the basis of our current findings, we depict a model that mild oxidative stress reduces NRF2 SUMOylation to promote KLK LUAD migration and invasion (Figure 5). Mild oxidative stress reduces NRF2 SUMOylation, which promotes KLK LUAD cell migration and invasion. Mechanistically, NRF2 SUMOylation increases the antioxidant ability of NRF2 and reduces cellular ROS levels, mainly by transcriptionally activating *Cat* in KLK LUAD cells. With reduced NRF2 SUMOylation, increased ROS act as signaling molecules to activate the JNK/c-Jun axis, which enhances cell mobility and cell adhesion, to promote LUAD cell migration and invasion.

## Data Availability

The data used to support the findings of this study are available from the corresponding author upon request.

## Supplementary Materials

Supplementary Materials Supplemental Experimental Procedures. Supplementary Table S1: primers used for PCDH-His-SUMO1 construction. Supplementary Table S2: primers for ChIP assay. Figure S1: NRF2 inhibits KLK LUAD cell migration and invasion independent of BACH1. Figure S2: NRF2 SUMOylation maintains KLK LUAD tumorigenesis and has no effect on KLK LUAD cell apoptosis. Figure S3: volcano plots of RNA-Seq results and GPX2 protein expression in four stable cell lines derived from A549 cells. Figure S4: NRF2 SUMOylation reduces ROS level in H2122 cells *via* transcriptional activation of *Cat* and inhibits migration and invasion through JNK/c-Jun axis.
