# Supplementary material for: Mild Oxidative Stress Reduces NRF2 SUMOylation to Promote Kras/Lkb1/Keap1 Mutant Lung Adenocarcinoma Cell Migration and Invasion
Source: Oxid Med Cell Longev. 2020 Nov 24;2020:6240125. doi: 10.1155/2020/6240125 (PMC7708001; doi:10.1155/2020/6240125)
Supplement: Supplementary Materials — Supplemental Experimental Procedures. Supplementary Table S1: primers used for PCDH-His-SUMO1 construction. Supplementary Table S2: primers for ChIP assay. Figure S1: NRF2 inhibits KLK LUAD cell migration and invasion independent of BACH1. Figure S2: NRF2 SUMOylation maintains KLK LUAD tumorigenesis and has no effect on KLK LUAD cell apoptosis. Figure S3: volcano plots of RNA-Seq results and GPX2 protein expression in four stable cell lines derived from A549 cells. Figure S4: NRF2 SUMOylation reduces ROS level in H2122 cells via transcriptional activation of Cat and inhibits migration and invasion through JNK/c-Jun axis. [file 6240125.f1.docx]

**Supplemental Experimental Procedures**

*Colony formation assay*

Cells (400–800 per well) were re-suspended in 6-well plates, and the medium was replaced every 3 days. After 10–14 days, colonies were fixed with 4% paraformaldehyde and then stained with crystal violet. Images of the colonies were taken using a scanner (Epson Perfection V800 Photo). The colonies were counted using ImageJ software and analyzed by GraphPad Prism.

*Annexin V and propidium iodide staining assay*

Cells were digested by trypsin solution without EDTA (Beyotime) and washed with PBS. Cells were then washed with 1x binding buffer, re-suspended in 100 µl of 1x binding buffer, and then stained with 5μl Annexin V and 2.5 µl propidium iodide staining solution for 15 min at room temperature. The apoptosis of cells was analyzed by flow cytometry (Becton Dickinson) immediately.

**Supplemental Tables**

Supplementary Table S1. Primers used for PCDH-His-SUMO1 construction

|  | 5’-3’ sequence |
| --- | --- |
| SUMO1-F | ATGTCTGACCAGGAGGCAAAACC |
| SUMO1-R | CTAAACTGTTGAATGACCCCCCGTT |
| His-SUMO1-F | ATGCATCACCATCACCATCACATGTCTGAC |
| XbaI-His-SUMO1-F | CTAGTCTAGAGCCGCCATGCATCACCATCACCATCAC |
| BamHI-His-SUMO1-R | CGCGGATCCCTAAACTGTTGAATGACCCCCCGTT |

Supplementary Table S2. Primers for ChIP assay

| ChIP-*Cat* promoter-F1 | CACCCAGCAGGGTCTAAGTAT |
| --- | --- |
| ChIP-*Cat* promoter-R1 | TCTGGCCCAGCAATTGGAGA |

**Supplemental Figures**

**
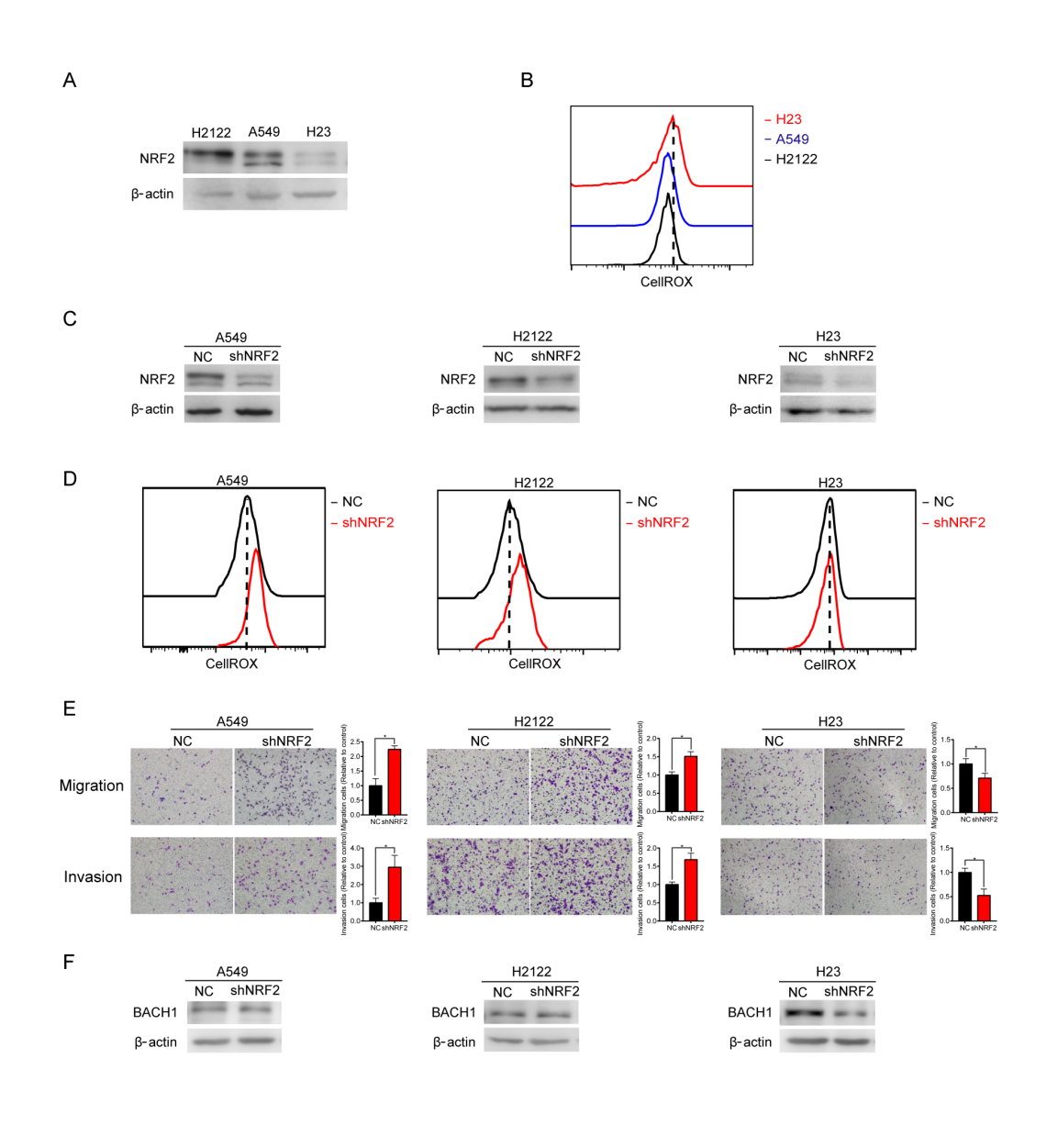
**

**Fig. S1** NRF2 inhibits KLK LUAD cell migration and invasion independent of BACH1.

**(A)** NRF2 expression in two KLK LUAD cell lines, A549 and H2122, and one KL LUAD cell line, H23. **(B)** Intracellular ROS levels in A549, H2122, and H23 cells. **(C)** NRF2 knockdown in A549, H2122, and H23 cells. **(D)** NRF2 knockdown increased intracellular ROS levels significantly in A549 and H2122 cells, but had no effect on the ROS level in H23 cells. **(E)** NRF2 knockdown promoted A549 and H2122 cell migration and invasion, but reduced H23 cell migration and invasion. **(F)** BACH1 expression in A549, H2122, and H23 cells after NRF2 knockdown.

**
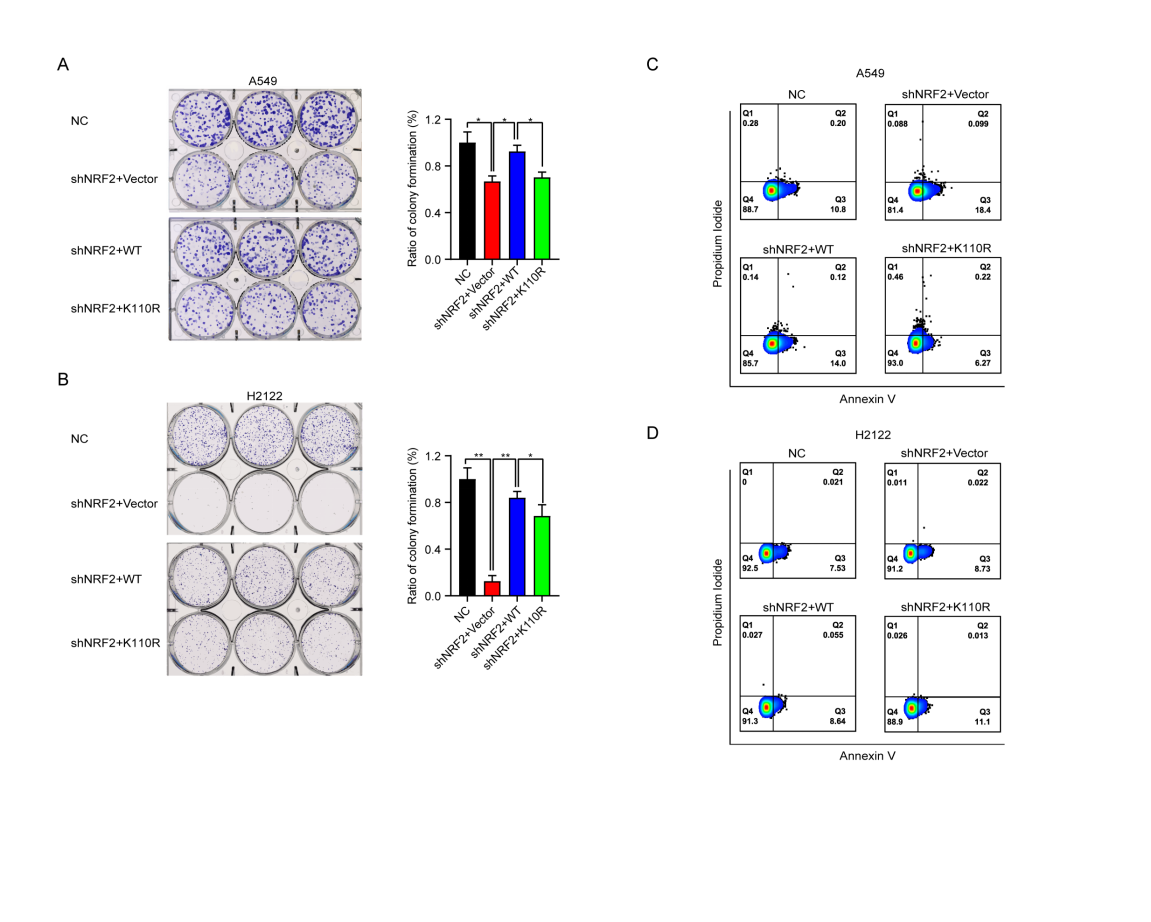
**

**Fig. S2** NRF2 SUMOylation maintains KLK LUAD tumorigenesis and has no effect on KLK LUAD cell apoptosis.

**(A)** Colony formation rate in four stable cell lines derived from A549 cells (3 replicates per group). **(B)** Colony formation rate in four stable cell lines derived from H2122 cells (3 replicates per group). **(C)** Apoptosis of four stable cell lines derived from A549 cells as measured by flow cytometry after Annexin V and propidium iodide staining. **(D)** Apoptosis of four stable cell lines derived from H2122 cells measured by flow cytometry after Annexin V and propidium iodide staining. *P<0.05, **P<0.01, and ***P<0.001.

**
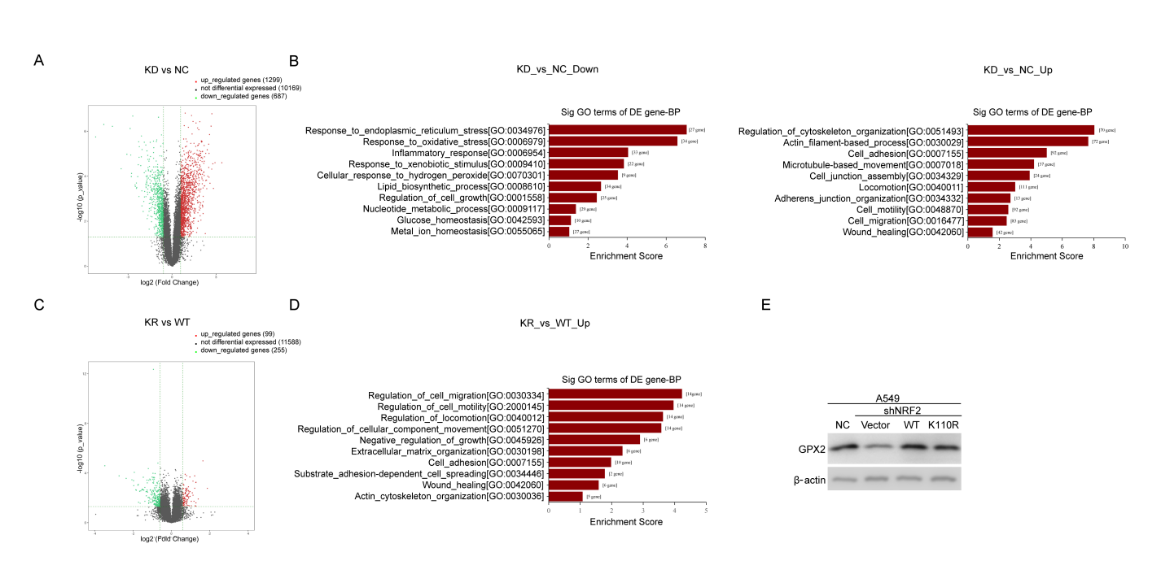
**

**Fig. S3** Volcano plots of RNA-Seq results and GPX2 protein expression in four stable cell lines derived from A549 cells.

**(A)** Volcano plot comparing gene expression in A549 KD vs A549 NC cells. **(B)** GO analysis (BP, biological process) of pathways differentially regulated in A549 KD vs A549 NC cells. **(C)** Volcano plot comparing gene expression in A549 KR vs A549 WT cells. **(D)** GO analysis (BP, biological process) of pathways differentially regulated in A549 KR vs A549 WT cells. **(E)** GPX2 protein expression in four stable cell lines derived from A549 cells.

**
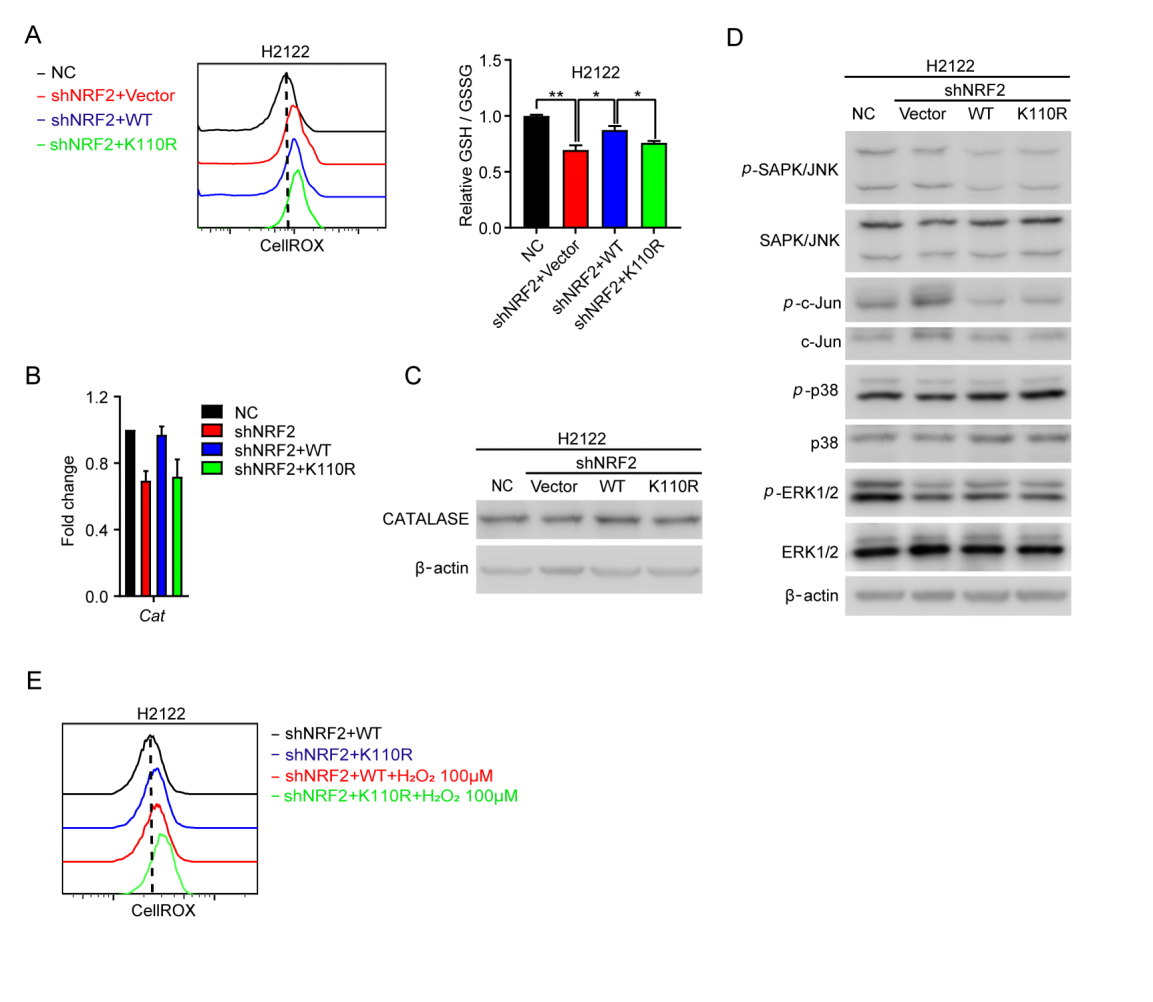
**

**Fig. S4** NRF2 SUMOylation reduces ROS level in H2122 cells *via* transcriptional activation of *Cat* and inhibits migration and invasion through JNK/c-Jun axis.

**(A)** The intracellular ROS level and GSH/GSSG ratio in four stable cell lines derived from H2122 cells (3 replicates per group). **(B)** The mRNA level of *Cat* in four stable cell lines derived from H2122 cells (3 replicates per group). **(C)** Catalase protein expression in four stable cell lines derived from H2122 cells. **(D)** Altered activation of MAPK signaling pathways (ERK, JNK and p38 signaling pathways) in four stable cell lines derived from H2122 cells. **(E)** H2122-shNRF2+WT and H2122-shNRF2+K110R cells were treated with 100 μΜ H_2_O_2_ for 12 h, and the intracellular ROS level was then measured by flow cytometry. *P<0.05, **P<0.01, and ***P<0.001.
